# Differential expression of immunoregulatory cytokines in adipose tissue and liver in response to high fat and high sugar diets in female mice

**DOI:** 10.3389/fnut.2023.1275160

**Published:** 2023-11-03

**Authors:** Juliane Weiner, Sebastian Dommel, Claudia Gebhardt, Martha Hanschkow, Yulia Popkova, Kerstin Krause, Nora Klöting, Matthias Blüher, Jürgen Schiller, John T. Heiker

**Affiliations:** ^1^Medical Department III - Endocrinology, Nephrology, Rheumatology, University of Leipzig Medical Center, Leipzig, Germany; ^2^Helmholtz Institute for Metabolic, Obesity and Vascular Research (HI-MAG) of the Helmholtz Zentrum München at the University of Leipzig and University Hospital Leipzig, Leipzig, Germany; ^3^Institute for Medical Physics and Biophysics, Medical Faculty, University of Leipzig, Leipzig, Germany; ^4^Deutsches Zentrum für Diabetesforschung e.V., Neuherberg, Germany; ^5^Institute for Biochemistry, Faculty of Life Sciences, University of Leipzig, Leipzig, Germany

**Keywords:** adipose tissue, obesity, inflammation, high fat diet, high sugar diet, lipid profile, nutrient, nutrition

## Abstract

A comprehensive understanding of how dietary components impact immunoregulatory gene expression in adipose tissue (AT) and liver, and their respective contributions to metabolic health in mice, remains limited. The current study aimed to investigate the metabolic consequences of a high-sucrose diet (HSD) and a high-fat diet (HFD) in female mice with a focus on differential lipid- and sucrose-induced changes in immunoregulatory gene expression in AT and liver. Female C57BL/6 J mice were fed a purified and macronutrient matched high fat, high sugar, or control diets for 12 weeks. Mice were extensively phenotyped, including glucose and insulin tolerance tests, adipose and liver gene and protein expression analysis by qPCR and Western blot, tissue lipid analyses, as well as histological analyses. Compared to the control diet, HSD- and HFD-fed mice had significantly higher body weights, with pronounced obesity along with glucose intolerance and insulin resistance only in HFD-fed mice. HSD-fed mice exhibited an intermediate phenotype, with mild metabolic deterioration at the end of the study. AT lipid composition was significantly altered by both diets, and inflammatory gene expression was only significantly induced in HFD-fed mice. In the liver however, histological analysis revealed that both HSD- and HFD-fed mice had pronounced ectopic lipid deposition indicating hepatic steatosis, but more pronounced in HSD-fed mice. This was in line with significant induction of pro-inflammatory gene expression specifically in livers of HSD-fed mice. Overall, our findings suggest that HFD consumption in female mice induces more profound inflammation in AT with pronounced deterioration of metabolic health, whereas HSD induced more pronounced hepatic steatosis and inflammation without yet affecting glucose metabolism.

## Introduction

The increasing prevalence of obesity and associated metabolic disorders has become a global health concern (1). Obesity causes chronic inflammation in adipose tissue and liver, which contribute to the development of local and systemic insulin resistance and finally type 2 diabetes (2) as well as non-alcoholic fatty liver disease and steatohepatitis (3).

Adipose tissue, apart from its role in energy storage, plays a pivotal role in the regulation of metabolic homeostasis through the secretion of adipokines and the modulation of immune responses (4). Excessive lipid accumulation in adipocytes results in hypertrophy and substantially alters the profile of adipose tissue secreted adipokines, from anti-inflammatory and insulin sensitizing to promoting inflammation and insulin resistance (5). Similarly, the liver serves as a central organ in metabolic regulation, lipid metabolism, and glucose homeostasis (3). AT and liver inflammatory signatures furthermore respond differently to weight loss (6). Therefore, understanding the effects of different dietary compositions on gene expression patterns in liver and adipose tissue is crucial to better understand the underlying mechanisms governing metabolic disorders.

Diet-induced metabolic disturbances have been extensively studied, particularly the effects of high-sucrose and high-fat diets as well as combinations thereof termed cafeteria or Western diets (7). Dietary changes relating to differences in the source of fat and carbohydrates as well as lipid composition have profound effects on the extent of diet-induced obesity and metabolic health. High-fat diets robustly induce weight gain with significant accumulation of adipose tissue, development of insulin resistance, hypertension, dyslipidemia, and metabolic syndrome (7). Effects of high-carbohydrate diets show more variability with respect to body weight gain and adipose tissue expansion, but also have notable negative metabolic effects in male mice and rats (8, 9). On the other hand, a very recent study does not find negative consequences of a life-long high-sucrose diet at all (10). Moderate HSD (up to 25% kcal from sucrose) did not have effects on body weight, AT expansion and metabolic parameters in male mice of the generally obesity prone C57BL/6 J background. In addition to the genetic background affecting the metabolic response to dietary fat or sugar (11), also sex-dependent metabolic adaptations in AT and liver control the initiation, progression and finally severity of metabolic diseases such as non-alcoholic fatty liver (NAFLD) (12). For example, under HSD, in addition to AT-derived lipids, the contribution of *de novo* lipogenesis to hepatic lipid deposition and NAFLD development seems more pronounced in female mice and independent from diet-induced hepatic or systemic insulin resistance (12).

A comprehensive understanding of how the diet composition impacts on immunoregulatory gene expression in adipose tissue (AT) and liver, and their respective contributions to metabolic health in mice, remains limited. It is important to note, that in obesity research, it is common to find sex differences rather than similarities. Unfortunately, there is a significant bias toward using male rodents in preclinical studies, as they are often preferred due to more pronounced disease phenotypes. However, as obesity and type 2 diabetes affect both men and women in the human population, it is also crucial to include females in research to improve their translational potential.

The present study aimed to address this knowledge gap by investigating the effects of matched high-sucrose diet (HSD) and high-fat diet (HFD) on metabolic health and gene expression profiles in AT and liver of female mice. We extensively phenotyped mice fed matched control (abbreviated as REF in figures), HFD and HSD for 12 weeks, to evaluate changes in body weight and body composition, glucose, and insulin tolerance, and conducted AT and liver gene and protein expression analyses. Additionally, lipid and histological analyses were performed to gain insights into alterations in lipid composition and the presence of hepatic steatosis in both HSD- and HFD-fed mice.

Based on previous research in male mice, we hypothesized that HFD consumption would induce more pronounced inflammation in AT, leading to significant deterioration in metabolic health, particularly glucose intolerance and insulin resistance (13, 14). In contrast, we anticipated that HSD consumption would lead to ectopic lipid deposition and substantial hepatic steatosis and inflammation, without yet affecting glucose metabolism (15, 16).

## Methods

### Animals

Female C57BL/6NTac mice (from Taconic Europe, Lille Skensved, Denmark) were group-housed (5 mice per cage) at 22°C with a 12 h light/dark cycle and *ad libitum* access to water and food. At 12 weeks of age, mice were assigned to three groups (*n* = 10 per group) of matched body weights to be fed either a low glycemic reference diet (REF; D12450J, 10 kJ% fat), a high fat diet (HFD; D12492, 60 kJ% fat) or a high sucrose diet (HSD; D12450B, 10 kJ% fat, 63% sucrose) for 12 weeks; the detailed compositions of the diets were previously published (17). Diets were manufactured by Ssniff (Ssniff GmbH, Soest, Germany). All animal experiments were approved by the local authorities of the Free State of Saxony (Landesdirektion Leipzig, Germany, TVV39/14), as recommended by the responsible local animal ethics review board.

### Mouse phenotyping

Intraperitoneal (i.p.) glucose tolerance tests (GTT) and insulin tolerance tests (ITT) were performed after 10 weeks on the respective diets. In brief, GTT was performed after an overnight fast of 12 h by i.p. injecting 2 g glucose per kg body weight. For ITT, 0.75 U of insulin per kg body weight were i.p. injected in *ad libitum* fed mice. Blood samples for glucose measurements were taken at different time points (0, 15, 30, 60, and 120 min post injection). Blood glucose was measured in whole-venous blood samples from fed mice using the FreeStyle Mini automated glucose monitor (Abbott GmbH, Ludwigshafen, Germany). Rectal body temperature was measured using a special probe (TH-5 Thermalert Clinical Monitoring Thermometer, Physitemp Instruments). Body composition (water and fat content) was determined in conscious mice by nuclear magnetic resonance technology using an EchoMRI-700 instrument (Echo Medical Systems, Houston, TX) at the beginning and every 3 weeks until the end of the study. Body weight and food intake (per cage) were measured weekly. Metabolic efficiency [energy intake (kJ)/energy stored in fat (kJ); as in von Essen et al. (18)] and feed efficiency [weight gain (g)/energy intake (kcal)] were calculated using average food intake per mouse for each individual cage. Serum insulin (#10–1,113-01, Mercodia), C-peptide (80-CPTMS-E01, ALPCO) and leptin (#90030, ChrystalChem) were determined by standard ELISA. Homeostatic model assessment of insulin resistance (HOMA-IR) was assessed using the following formula: [fasting glucose (mmol/L) × fasting insulin (μU/mL)/22.5]. After 12 weeks on the respective diets, mice were sacrificed by CO_2_ inhalation after overnight fasting and organs were immediately removed, weighed (liver, subcutaneous inguinal (iWAT), gonadal (gWAT) and interscapular brown adipose tissue (BAT) and frozen in liquid nitrogen).

### Adipose tissue characterization

AT and liver tissues were fixed in 4% paraformaldehyde (pH 7.4) for 24 h at 4°C and subsequently embedded in paraffin. Tissue slices were stained using Hematoxylin–Eosin (HE) and digital images were obtained using a Keyence BZ-X810 microscope (Keyence; Osaka; Japan). Adipocyte lipid droplet sizes were measured by automated analysis of histological sections using Image J and the open-source plugin Adiposoft as previously reported (19). Three pictures were evaluated, and 300 adipocytes were counted per sample; objects with a diameter below a pre-defined threshold (25 μm) were removed from each image.

### Lipid analyses

Lipid extraction from adipose and liver tissues was performed according to Matyash et al. (20) and triglycerides (TG) as well as phospholipids were analyzed by MALDI-TOF MS as essentially described in (21, 22). All MALDI experiments were performed on a Bruker Autoflex mass spectrometer. Either 2,5-dihydroxybenzoic acid (DHB, 0.5 M in methanol) or 9-aminoacridine (9-AA, in isopropanol/acetonitrile (60:40 v/v)) was used (22) to record positive and negative ion spectra, respectively. DHB was used to analyze the TG and the phosphatidylcholines (PC) composition (in the positive ion mode) while 9-AA (in the negative ion mode) was used to evaluate the phosphatidylethanolamines (PE) species (23). Spectra were recorded in the reflector mode to decrease the peak-widths. No dedicated baseline correction was necessary. The intensities of all species (using FlexAnalysis software by Bruker, Bremen, Germany) of the respective lipid class were added and all data are given (in %) with reference to this value. Relative data are given because we did not add internal standards, which is a prerequisite for absolute data. As the mass spectrometer used has a rather limited dynamic range, where detector responses directly correlate with analyte concentrations, the addition of an internal standard is challenging because of the need to avoid adding an excess of the internal standard. Therefore, only relative (but not absolute) data can be given. This method has been used successfully in the past (22). Data obtained by electrospray ionization (ESI MS) in combination with thin-layer chromatography (TLC) (as essentially described in (24)) gave identical results. We have focused on these lipid classes as TG constitute about 98% of all the lipids, and the complete removal of TG is very challenging and aggravates the analysis of all further lipid species. We did not intend to perform a comprehensive “lipidomics” study (with the detection of “all” lipids) but focused on the most important and abundant lipid classes. Total liver triglycerides (TGs) and cholesterol were determined in tissue lysates using commercial assays according to the manufacturers’ instructions (#290–63,701, #294–65,801, Wako Diagnostics).

### Western blot analyses

Western blot analysis was done for samples of AT (iWAT, gWAT and BAT) and liver as previously described (25). Briefly, after extraction in RIPA buffer (150 mM NaCl, 10 mM Tris pH 7.2, 0.1% SDS, 1% Triton X-100, 1% sodium deoxycholate, and 5 mM EDTA, complemented with protease- and phosphatase inhibitors), proteins were separated by SDS-PAGE and Western blotting was performed using the tank blot method. The following antibodies and dilutions were used: from Cell Signaling Technologies, Danvers, MA, USA: FAS (#3189S), HSL (4107S), pHSL (4126S), anti-rabbit-HRP (#7074), anti-mouse-HRP (#7076); from Sigma-Aldrich, St. Louis, MO, USA: ACTB (#A1978). Chemiluminescence was detected using the G:BOX Chemi XX9 documentation system with GeneTools analysis software (Syngene, Cambridge, UK).

### Quantitative real-time-PCR (qPCR)

RNA isolation and quantitative real-time PCR were performed as previously described (25). All primer sequences are listed in Supplementary Table S1. mRNA expression of selected candidate genes reflecting key AT and liver functions was calculated relative to *36b4* (26, 27) using the ΔΔCT method.

### Statistical analyses

Data are presented as means ± SEM. Statistical analyses were performed using GraphPad Prism 9 (GraphPad, San Diego, CA, USA). Methods of statistical analyses were chosen based on the design of each experiment and are indicated in the figure legends. Adjusted *p* < 0.05 was considered statistically significant.

## Results

### Weight gain, food intake, body temperature and composition

**Figure 1:**
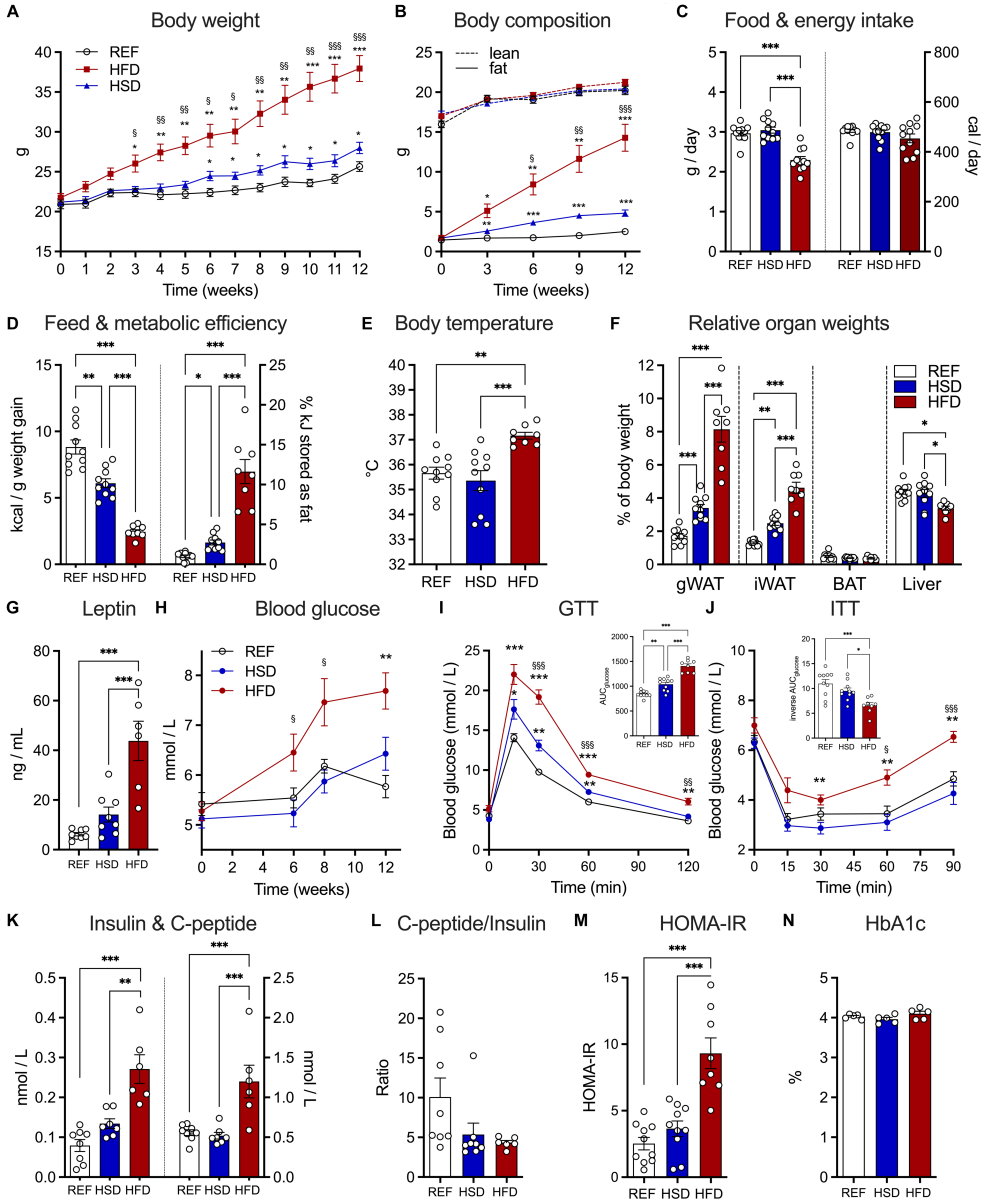
Development of diet-induced obesity and effects on glucose metabolism in response to 12 weeks of HSD and HFD feeding. **(A)** Body weight development in HSD- and HFD-fed and control mice during the 12-week study. **(B)** Absolute body fat (solid lines) and lean mass (dotted lines) were determined using EchoMRI at week 0, 3, 6, 9, and 12. **(C)** Food intake (left) and total energy intake (right) as well as **(D)** feed (left) and metabolic efficiency (right). **(E)** Core body temperatures, **(F)** relative organ weights of iWAT, gWAT, BAT and liver and **(G)** leptin levels in serum of HSD- and HFD-fed and control mice at the end of the study. **(H)** Fed state blood glucose levels in HSD- and HFD-fed and control mice over the course of the study. **(I,J)** Intraperitoneal glucose tolerance test (GTT; **I**) and insulin tolerance tests (ITT, **J**). Inserts show calculated areas under the curve. **(K)** Serum insulin (left) and C-peptide (right) levels at the end of the study and **(L)** resulting C-peptide to insulin ratio. **(M)** Calculated HOMA-IR and **(N)** measured HbA1c at the end of the study. Color coding for all graphs: black - control diet; blue - HSD, red - HFD. Data are presented as mean ± SEM, with *N* = 6–8 mice per group. Statistical significance was tested by two-way ANOVA with Holm-Šídák’s **(A,B)** or Tukey’s **(F,H–J)** multiple comparisons test, or by ANOVA with Tukey’s multiple comparisons test and is indicated as follows: *, vs. REF; ^§^ HSD vs. HFD; *, ^§^ *p* < 0.05; **, ^§§^ *p* < 0.01; ***, ^§§§^ *p* < 0.001.

HFD-fed mice exceeded weight gain of REF- and HSD-fed mice and had significantly higher body weights after 3 weeks on the diet (Figure 1A). The average end of study body weight of HFD-fed mice was >30% higher compared to mice fed the other diets (REF: 25.7 ± 0.6 g; HSD: 28.0 ± 0.7 g; HFD: 38.0 ± 1.6 g). Body weights of HSD-fed mice began to exceed REF-fed mice after 7 weeks on the diets (Figure 1A) and were significantly heavier at the end of the study (Figure 1A). EchoMRI analyses demonstrated the significant accumulation of body fat in HFD-fed mice (14.3 ± 1.7 g at the end of study), compared to the REF- (2.5 ± 0.2 g) and HSD-fed mice (4.8 ± 0.4 g; Figure 1B), while lean body mass was not altered by the diets (Figure 1B). Food consumption was significantly lower in HFD-fed mice (Figure 1C, left), but overall energy intake was similar with all diets (Figure 1C, right), demonstrating the significantly greater feed efficiency (kcal / g weight gain) of both HSD and HFD compared to the REF diet (Figure 1D, left), and the significantly higher metabolic efficiency (% kJ consumed per kJ stored as fat) of mice consuming a HFD and to a lesser extent also HSD, compared to REF-fed controls (Figure 1D, right). HFD-fed mice also exhibited significantly higher core body temperatures, compared to REF- and HSD-fed mice (Figure 1E). Relative WAT depot weights (as % of body weight) were significantly higher in HFD (four-fold) and to lesser extent also in HSD-fed mice (two-fold) compared to REF-fed controls (Figure 1F). Relative BAT weight was not affected by diets and relative liver weight was significantly reduced in HFD-fed mice (Figure 1F). Reflective of the increased fat mass, leptin serum levels were significantly higher in HFD-fed mice (41.3 ± 6.2 ng/L) and also increased in HSD-fed mice (14.1 ± 3.0 ng/L) without reaching statistical significance when compared to respective controls (6.2 ± 0.7 ng/L) (Figure 1G).

### Serum parameters, insulin, and glucose tolerance

After 6 weeks on the respective diets, blood glucose levels of HFD-fed mice were already significantly increased, while those of REF- and HSD-fed mice where not different, even after 12 weeks on the diets (Figure 1H). As expected, glucose tolerance was strongly impaired in HFD-fed mice, but also significantly reduced in HSD-fed mice compared to REF-fed controls (Figure 1I). In glucose tolerance tests, the area under the curve was increased by *ca*. 75% in HFD- and *ca*. 25% in HSD-fed mice compared to controls (Figure 1I, insert). Similarly, insulin sensitivity was significantly impaired in HFD-fed mice, while HSD-fed mice still had comparable insulin sensitivity compared to controls (Figure 1J). Pronounced fasting hyperinsulinemia was observed in HFD-fed mice, with significantly higher insulin (three-fold compared to REF, Figure 1K, left) and C-peptide levels (two-fold compared to REF; Figure 1K, right). Insulin levels were also higher in HSD-fed mice, but not statistically significant (Figure 1K). Yet, the C-peptide to insulin ratio, as a marker of insulin clearance, was significantly decreased in both, HFD- and HSD-fed mice (Figure 1L). In line, the HOMA-IR index indicated pronounced insulin resistance specifically in HFD-fed mice (Figure 1M), yet HbA1c as a marker of long-term hyperglycemia was not different (Figure 1N).

### Diet-induced changes in WAT adipocyte morphology and TG composition

**Figure 2:**
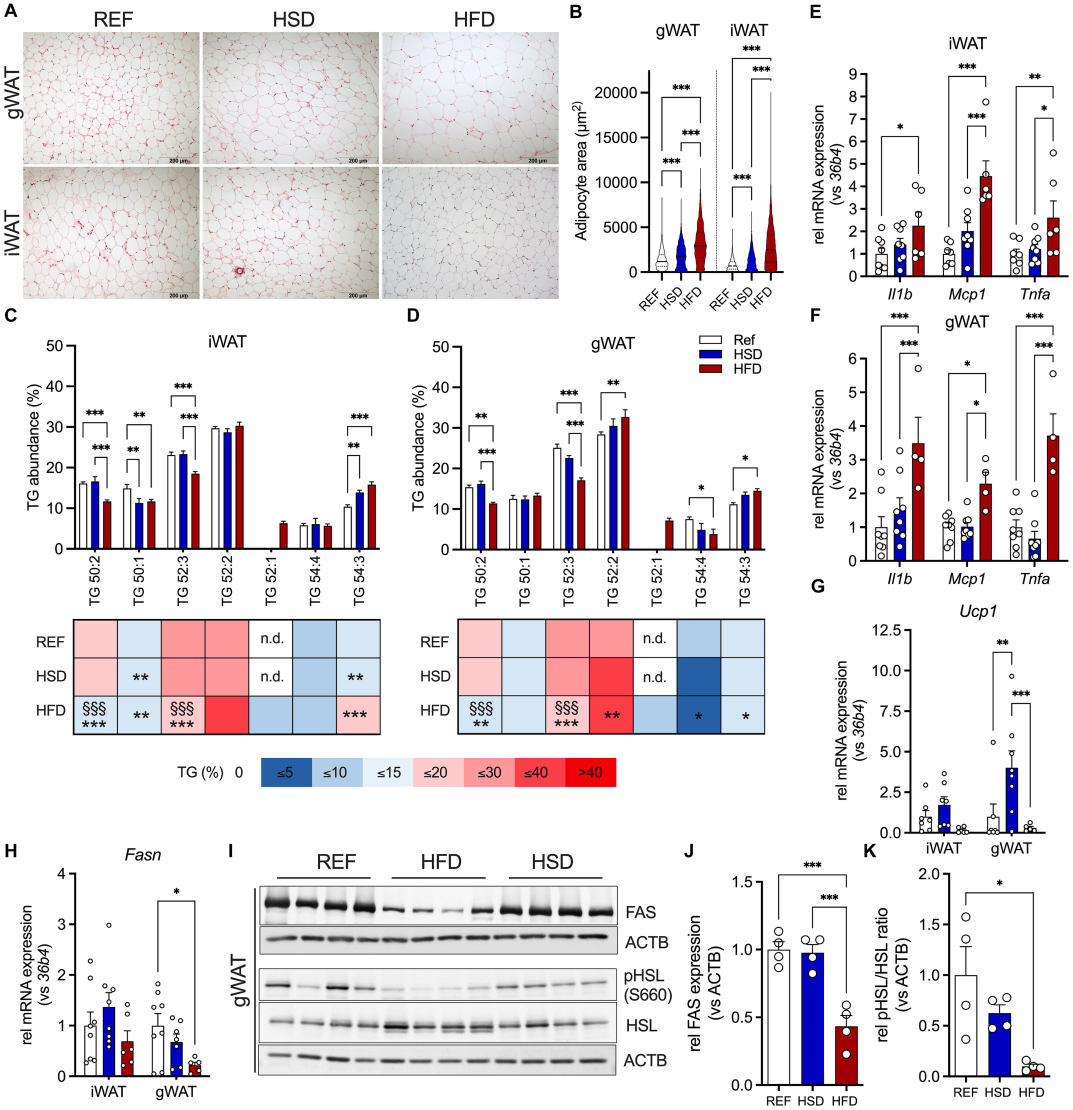
Adipocyte hypertrophy, inflammation, and lipid composition in WAT depots in response to 12 weeks of HSD and HFD feeding. **(A)** Representative hematoxylin and eosin (H&E) sections of inguinal (iWAT, top) and gonadal white adipose tissue (gWAT, bottom) from HSD- and HFD-fed mice compared to controls. **(B)** Adipocyte sizes determined in iWAT and gWAT H&E stainings. **(C,D)** Relative abundances of triglycerides (TG) in iWAT and gWAT of HFD- and HSD-fed and control mice at the end of the study. **(E–H)** The expression of inflammatory genes *Il1b*, *Mcp1* and *Tnfa* in iWAT **(E)** and gWAT **(F)**, as well as expression of *Ucp1* **(G)** and *Fasn* **(H)** in both iWAT (left) and gWAT (right). **(I)** Western blot analysis of HSL phosphorylation (pHSL (Ser660) and HSL) as well as FAS expression in gWAT (*n* = 4/group). **(J,K)** Densitometric analyses of HSL-phosphorylation **(J)** and FAS expression **(K)**, both normalized for bActin expression. Color coding for all graphs: black - control diet; blue - HSD, red - HFD. Data are presented as mean ± SEM, with *N* = 6–8 mice per group. Statistical significance was tested by two-way ANOVA with Tukey’s **(C–F)** multiple comparisons test or uncorrected Fischer’s LSD **(G,H)**, or by ANOVA with Tukey’s **(B,J,K)** multiple comparisons test and is indicated as follows: *, vs. REF; ^§^ HSD vs. HFD; *, ^§^ *p* < 0.05; **, ^§§^ *p* < 0.01; ***, ^§§§^ *p* < 0.001.

Both HFD and HSD-fed mice presented significantly larger adipocytes in gWAT (Figure 2A, top) and iWAT (Figure 2A, bottom). Adipocyte hypertrophy (100% increase in adipocyte area) was only observed in HFD-fed mice, while adipocytes of HSD-fed mice were ≈30% larger compared to controls (Figure 2B). We further investigated triglyceride (TG) composition of AT depots, to assess the relative abundances of TG-species in WAT (Figures 2C,D). The main TG present in both WAT depots, TG 52:2, was comparable between all groups and constituted ≈30% of all TG in WAT. Shorter chain fatty acid (FA) containing TGs 50:1 and 52:3 were significantly reduced in HFD-fed mice compared to REF- and HSD-fed mice, while longer chain FA-containing TG 54:3 was higher in both HSD and HFD- fed mice. TG 52:1 was only detected in WATs of HFD-fed mice. Together, the HFD affected both, WAT adipocyte size and triglyceride composition, to a higher extent than the HSD.

### Diet-induced changes in WAT inflammation, browning and lipid metabolism

We then investigated diet-induced changes in inflammatory (*Il1b, Mcp1, Tnfa*), metabolic (*Fasn, Glo1*) and thermogenic (*Cox7a1, Tbx1, Tmem26, Ucp1*) genes in WAT depots. The expression of immunoregulatory cytokines *Il1b, Mcp1* and *Tnfa* was significantly induced in HFD-fed mice in both WAT depots compared to REF- and HSD-fed mice (≈2-4-fold; Figures 2E,F). We further measured gene expression related to WAT browning and thermogenesis. The expression of UCP1, the main driver of thermogenesis in brown adipoctyes, showed diet-dependent regulation. While in gWAT, expression of *Ucp1* was significantly increased in HSD-fed mice compared REF-fed controls, the HFD significantly suppressed *Ucp1* expression in both WAT depots (Figure 2G). Gene expression of other markers of AT WAT browning (such as *Cox7a1*, *Tbx1* and *Tmem26*) were not affected by the diets, except for gWAT *Tmem26*, which was also significantly reduced in HFD-fed mice, in line with a reduction in *Ucp1* (Supplementary Figures S1A,B). Expression of methylglyoxal detoxifying *Glo1*, a key enzyme protecting against negative metabolic consequences of high dietary sucrose together with fatty acid synthase (Fasn), was not significantly different in all diet groups (Supplementary Figures S1A,B). In gWAT, *Fasn* gene expression was significantly reduced in HFD-fed mice (Figure 2H), which translated into significantly reduced FAS protein levels, which was not observed in HSD-fed mice (Figures 2I,J). Also, HSL-phosphorylation was significantly reduced by ≈90% in HFD-fed mice (Figures 2I,K). Also in iWAT, a trend for decreased *Fasn* expression was observed in HFD-fed mice (Figure 2H), that translated into significantly reduced FAS expression in HFD- compared to HSD and control mice (Supplementary Figure S1C). Finally, phosphorylation of HSL was also increased in iWAT of HSD-fed mice compared to both other groups, without reaching statistical significance (Supplementary Figure S1C). In summary, the HFD induced robust expression of immunoregulatory cytokines in both WAT depots, while suppressing gene expression related to WAT browning and thermogenesis. In contrast, the HSD led to increased expression of *Ucp1* in both WAT depots, with increased FAS expression in iWAT.

### Diet-induced changes in BAT

**Figure 3:**
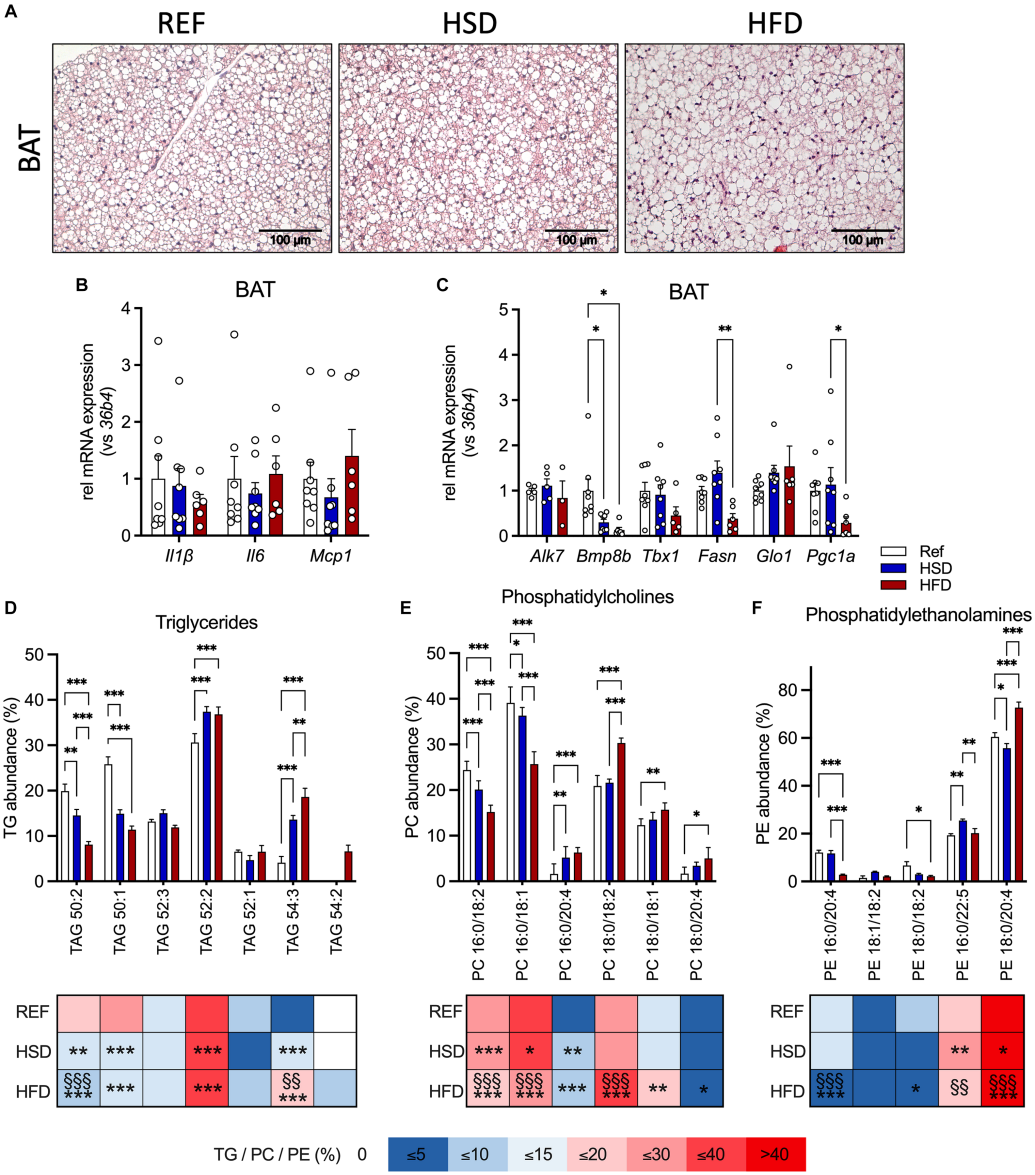
Adipocyte hypertrophy, inflammation, and lipid composition in BAT in response to 12 weeks of HSD and HFD feeding. **(A)** Representative hematoxylin and eosin (H&E) sections of brown adipose tissue (BAT) from HSD- and HFD-fed mice and controls. **(B)** The expression of inflammatory genes *Il1b*, *Il6* and *Mcp1*, as well as **(C)** expression of thermogenic genes (*Alk7, Bmp8b, Tbx1, Fasn, Glo1 and Pgc1a*) in BAT of HFD- and HSD-fed and control mice. **(D–F)** Relative abundances of triglycerides (TG; **D**), phosphatidylcholines (PC; **E**) as well as phosphatidylethanolamines (PE; **F**) in BAT at the end of the study. Color coding for all graphs: black - control diet; blue - HSD, red - HFD. Data are presented as mean ± SEM, with *N* = 6–8 mice per group. Statistical significance was tested by two-way ANOVA with Tukey’s comparisons test and is indicated as follows: *, vs. REF; ^§^ HSD vs. HFD; *, ^§^ *p* < 0.05; **, ^§§^ *p* < 0.01; ***, ^§§§^ *p* < 0.001.

BAT histology did not reveal obvious changes, with a mixed morphology consisting of uni- and multilocular adipocytes, though with enlarged lipid droplets, in line with intermediate BAT activation under the mild-cold stress of housing at room temperature of 22°C (Figure 3A). No significant changes in cytokine gene expression were found (Figure 3B). Investigating genes related to thermogenesis revealed that the HFD led to reduced expression of *Bmp8b*, *Pgc1a* and *Tbx1* (only in trend) compared to HSD and REF-fed mice (Figure 3C). In addition, *Fasn* expression was significantly reduced in BAT of HFD-fed mice (Figure 3B).

We also investigated the TG composition in BAT (Figure 3D), together with phosphatidylcholines (PC, Figure 3E) and phosphatidylethanolamines (PE, Figure 3F) as the most abundant phospholipids (as already outlined by Popkova et al., (22)). Our results indicate that TG 50:1 and TG 50:2 were significantly reduced in HSD- and HFD- fed mice compared to controls. Independent of the diet, TG 52:2 was the most abundant TG, with significantly higher levels in HSD- and HFD-fed mice. As observed in WAT, relative abundances of shorter chain FA containing TG 50:1 and TG 50:2 were significantly reduced (by 50%) in both HSD and HFD, while levels of longer chain FA containing TG 54:3 were significantly higher (by >300%). Levels of TG 52:3 were lower in BAT and not affected by diet, in contrast to WAT. TG 52:1 was present in BAT independent of diet and TG 54:2 was only detected in HFD-fed mice. For PC (Figure 3E), PC 16:0/18:2 and PC 16:0/18:1 were significantly reduced in HSD and HFD compared to HSD-fed and control mice. PC 16:0/20:4, PC 18:0/18:2, PC 18:0/18:1 and PC 18:0/20:4 were significantly elevated in HFD-fed compared to control mice. For PE (Figure 3F) we found, the abundant PE, PE 18:0/20:4, was significantly lower in HSD-fed, and significantly higher in HFD-fed compared to both HSD-fed and control mice. PE 16:0/20:4 was significantly reduced (>75%) in HFD-fed compared to HSD-fed and control mice. PE 18:0/18:2 was significantly reduced in HFD-fed compared to control mice, while PE 16:0/22:5 was significantly higher in HSD- and HFD-fed mice compared to controls. Our focus was on these three lipid classes because they represent the - by far - most abundant ones. Taken together, these results show distinct diet-induced changes in AT lipid composition: some are generally observed in white and brown AT, while others are depot-specific. Significant changes in HSD-fed mice compared to controls were found albeit identical quantity and composition of nutritional lipids are present in the matched diets.

In summary, we find that both diets significantly altered the BAT lipid composition without inducing inflammation in BAT.

### Diet-induced changes in liver

**Figure 4:**
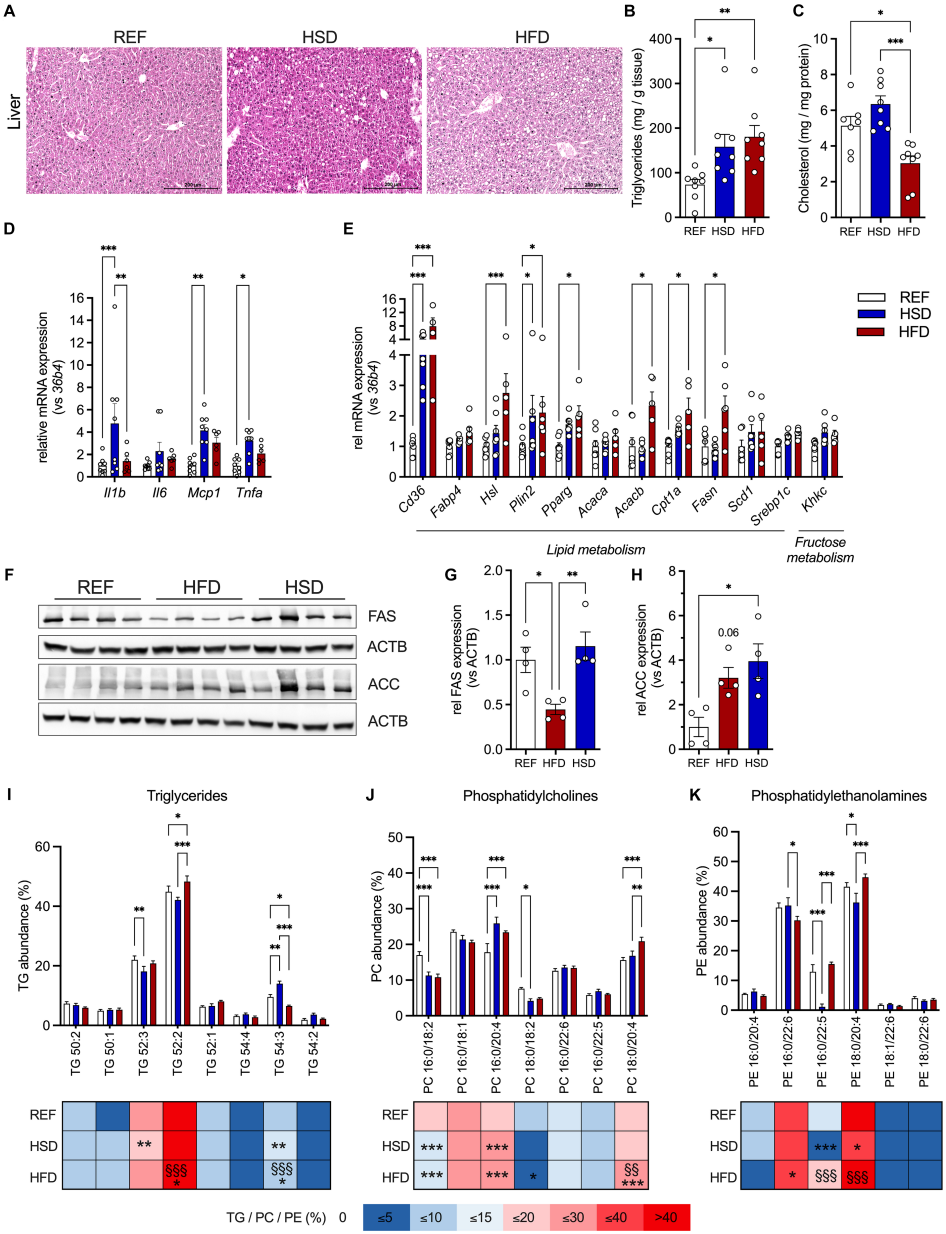
Ectopic lipid deposition, inflammation, and lipid composition in the liver in response to 12 weeks of HSD and HFD feeding. **(A)** Representative hematoxylin and eosin (H&E) stained liver sections of HFD- and HSD-fed and control mice. **(B,C)** Liver triglyceride (TG; **B**) and cholesterol content **(C)**. **(D)** The expression of inflammatory genes *Il1b*, *Il6*, *Mcp1* and *Tnfa*, as well as **(E)** expression of genes related to lipid metabolism (*Cd36, Fabp4, Hsl, Plin2 and Pparg*) and lipogenesis (*Acaca, Acacb, Cpt1a, Fasn, Scd1, Srebp1c*) and fructose metabolism (*Khkc*) in livers of HFD- and HSD-fed and control mice. **(F)** Western blot analysis of FAS and ACC expression in liver (*n* = 4/group). **(G,H)** Densitometric analyses of FAS **(G)** and ACC **(H)** expression, both normalized for ACTB expression. **(I-K)** Relative abundances of triglycerides (TG; **I**), phosphatidylcholines (PC; **J**) as well as phosphatidylethanolamines (PE; **K**) in the livers. Color coding for all graphs: black - control diet; blue - HSD, red - HFD. Data are presented as mean ± SEM, with *N* = 6–8 mice per group. Statistical significance was tested by ANOVA with Tukey’s **(B,C,G,H)** multiple comparisons test, or two-way ANOVA with Tukey’s **(D,I–K)** multiple comparisons test or uncorrected Fischer’s LSD **(E)** and is indicated as follows: *, vs. REF; ^§^ HSD vs. HFD; *, ^§^ *p* < 0.05; **, ^§§^ *p* < 0.01; ***, ^§§§^ *p* < 0.001.

Liver histology revealed the occurrence of ectopic lipid deposition in mice fed both HFD and HSD, but most pronounced in the HSD-fed mice (Figure 4A). Liver TG levels were significantly higher in HFD- (three-fold) and HSD-fed (2.5-fold) mice compared to control mice (Figure 4B). In contrast, liver cholesterol levels were significantly decreased (≈40%) in HFD-fed mice and trended higher (≈125%) in HSD-fed mice compared to the REF group (Figure 4C).

In contrast to WAT depots, we found inflammatory genes *Il1b*, *Mcp1* and *Tnfa* significantly increased in livers of HSD-fed animals (Figure 4D). Gene expression of key molecules related to hepatic lipid metabolism (*Cd36, Fabp4, Hsl, Plin2, Pparg, Acaca, Acacb, Cpt1, Fasn, Scd1 and Srepb1c*) revealed significant increase of *Cd36* and *Plin2* in both HFD- (≈8-fold and ≈2-fold, respectively) and HSD-fed (≈4-fold and ≈2-fold, respectively) mice (Figure 4E). Additionally, *Hsl*, *Pparg*, *Acacb*, *Cpt1a* and *Fasn* expression were significantly higher in HFD-fed mice (Figure 4E). In HSD-fed mice, liver expression of *Hsl*, *Pparg*, *Cpt1a*, *Scd1* and *Srebp1c* were also increased, though these changes did not reach statistical significance. Expression of the rate-limiting enzyme of fructose metabolism *Khkc* also showed a trend to be increased in HSD-fed mice (Figure 4E). As also observed in AT, FAS was decreased in livers of HFD-fed mice while ACC expression was significantly increased in livers of HSD-fed mice (Figures 4F–H). Liver lipid composition was characterized by more subtle diet-induced changes (Figures 4I–K). Here, 52:2 and 52:3 TGs were the major lipid constituents in livers independent of diet, and only livers of HSD-fed mice showed increased levels of longer chain FA containing TG 54:3 (Figure 4I). Regarding phospholipids, PC 16:0/18:2 and PC 18:0/18:2 were reduced by ≈33% in HSD- and HFD-fed mice, whereas PC 16:0/20:4 was elevated in HSD- and HFD-fed mice and PC 18:0/20:4 was higher in HFD-fed mice (Figure 4J). Major hepatic PEs were PE 16:0/22:6 which was reduced in HFD-fed mice, and PE 18:0/20:4 and PE 16:0/22:5 which were significantly reduced in HSD-fed mice (Figure 4K).

In summary, both energy-dense diets induced ectopic lipid deposition in the liver, but only livers of HSD-fed mice already showed significant induction of inflammatory gene expression.

## Discussion

In this study, we compared the effects of a high-fat diet (HFD) and a high-sucrose diet (HSD) on metabolic health in female mice. We observed an intermediate effect of the HSD on various parameters of obesity and insulin resistance, compared to HFD- and REF-fed mice. At the end of the 12-week diet, body weight was almost 10% higher in HSD-fed mice compared to REF-fed controls, whereas HFD-fed mice developed pronounced obesity with a 50% higher body weight. Both diets impaired glucose metabolism and insulin sensitivity, with the most detrimental effects observed in HFD-fed mice. Both HFD- and HSD-fed mice accumulated significantly more body fat with enlarged adipocytes compared to control mice, but only HFD-fed mice exhibited substantially increased body fat mass and adipocyte hypertrophy. While HFD resulted in pronounced obesity with a profound impact on metabolic health, clear indications of metabolic deterioration were already observed in HSD-fed mice despite a comparably small effect on body weight and composition at the end of the study.

We were further specifically interested in diet-induced changes in immunoregulatory gene expression in AT and liver. Together with infiltrating immune cells (28, 29), adipocytes express and secrete an array of inflammatory cytokines such as TNFa, MCP1, and IL6 (30–32). Serum levels of these cytokines are significantly increased in obese and insulin resistant individuals and drive metabolic diseases (33, 34). In AT, the HFD had more severe impact on immunoregulatory gene expression, especially in the gWAT, with all cytokines investigated significantly induced, including *Il1b*, *Mcp1*, and *Tnfa*. This was accompanied by the HFD-specific development of marked adipocyte hypertrophy. In contrast, neither energy-dense diet had significant effects on inflammatory gene expression in BAT.

In the liver, both the HSD and HFD increased hepatic lipid content with visible ectopic lipid deposition. However, the expression of immunoregulatory cytokines *Il1b*, *Mcp1*, and *Tnfa* was only significantly induced in HSD-, but not HFD-fed mice. The HSD led to hepatic gene expression patterns also observed in NAFLD despite <10% difference in body weight. The strong induction of hepatic CD36 likely represents a key mechanism promoting fatty liver disease by increasing uptake of free fatty acids (FFA) as well as expression of *de novo* lipogenic genes (35). In hepatocytes, as well as adipocytes and immune cells, CD36 is a key regulator of long-chain fatty acid uptake. Furthermore, adipose-derived CD36-containing exosomes can be endocytosed by hepatocytes, where they promote further lipid accumulation and inflammation. CD36 drives inflammation by also serving as a molecular pattern recognition receptor in immune and non-immune cells, inducing NF-κB pathways and the production of pro-inflammatory cytokines (36). Altered *Cd36* expression is associated with NAFLD, and directly contributes to the development of fatty liver under conditions of elevated FFA by modulating the rate of FA uptake by hepatocytes (37). In line, disruption of hepatic CD36 protected against NAFLD-associated systemic inflammation and insulin resistance, by reducing hepatic FFA uptake and ectopic lipid deposition in HFD-fed mice (37). In addition to long-chain FFA, CD36 also binds various extracellular inflammatory mediators (36). While we have not measured serum lipid profile in this study, the increased expression of *Cd36* in livers of HSD-fed mice may not only be a response to higher circulating lipids and FFA but is likely reflective of its role as signal transducer in immune cell activation and inflammation. The high sucrose intake further promotes lipogenesis and steatosis in the liver, due to increased fructose uptake, that is even further enhanced in the presence of glucose (38), and fructose metabolism providing additional DNL substrates (39, 40), and for example reflected by increased ACC expression in this study. The contribution of DNL in addition uptake of dietary and AT lipolysis-derived free fatty acids to the development of NAFLD has been shown to be specifically significant in female mice (12). Male mice under HSD rather showed preferential shuttling of DNL-derived lipids to the AT resulting in adipocyte hypertrophy. In the cell, fructose acts as an inducer of lipogenic gene expression and rapid phosphorylation by fructokinase C has been linked to ATP and phosphate depletion with subsequent uric acid generation, that may further drive NAFLD (41). In addition, further mechanisms have been reported that drive hepatic steatosis in response to high fructose intake (alone or as sucrose). Fructose has been shown to be efficiently metabolized in the intestine (by ubiquitous fructokinase A) and leads to increased gut permeability, and increased endotoxin passage and thereby trigger steatosis (42). Furthermore, fructose intake fosters alterations in the gut microbiome that likely further promote NAFLD progression (43).

Our analyses of relative abundances of the most abundant lipid species revealed significant impact of HSD and HFD. However, they do not allow the estimation of differences in total lipid abundance (nor of TG abundance, that were observed in liver via histology and TG measurements) due to the experimental limitation. Yet, we were more interested, whether the diets that provide the identical nutritional lipid source in lard may result in subtle differences in TG, PE and PC species, that in the liver may be explained by additional lipogenesis under HSD conditions. TG composition changes observed in WAT and BAT of HFD- and HSD-fed mice were mostly of the same direction and more pronounced in HFD-fed mice. But in liver, the HSD induced alterations were either more pronounced (e.g., TG52:3; PC16:0/20:4) or indicated an opposite direction (e.g., TG52:2, TG54:3; PE16:0/22:5, PE18:0/20:4) when compared to HFD-fed mice. Drivers of these changes were found in the differential expression of key lipogenic enzyme FAS and activation of lipolytic HSL in AT and liver. FAS catalyzes the synthesis of palmitic acid from acetyl-CoA and malonyl-CoA, whereas longer chain fatty acid production is dependent of ELOVL activities (44, 45). Confirming previous studies, showing reduced *de novo* fatty acid synthesis in obese mice (46) and humans (47), we found a 50% reduction on FAS protein levels in gWAT of HFD-fed mice. In contrast, FAS protein levels were higher in iWAT of HSD-fed mice compared to REF controls. In the liver, *Fasn* expression was not significantly different, though in trend induced in HFD-fed mice. HSL phosphorylation and activation was elevated in iWAT of HSD-fed mice, but reduced in gWAT under both HSD and HFD, suggesting a complex interplay between diet and the regulation of lipid metabolism in adipose tissue. Enhanced glycolysis under conditions of high levels of dietary sucrose intake results in increased production of methylglyoxal, a highly reactive α-oxoaldehyde derived from glycolysis through a non-enzymatic reaction. Glyoxalase 1 (Glo1) is the enzyme neutralizing methylglyoxal, and has been shown to protect against negative metabolic consequences of a HSD in a complex interplay with FAS-mediated FA synthesis (48). While increased FAS expression in iWAT seems to compensate reduced *Glo1* expression, the lower FAS expression in gWAT was not compensated by altered *Glo1* expression. This suggests that this system is differentially affected in AT depots of chronically HSD-fed mice and indicates dysfunction of this rescue mechanism in gWAT.

In conclusion, we show that HFD predominantly affected AT inflammation, while the HSD induced inflammatory gene expression in the liver, without affecting AT. High-sugar diets have been linked to increased DNL and development of NAFLD in humans, also without significant weight gain (49). Consequently, reduced dietary sucrose intake has been proposed as a strategy to treat NAFLD and has been shown to limit hepatic DNL and significantly lower liver fat (50, 51).

## Data availability statement

The original contributions presented in the study are included in the article/supplementary material, further inquiries can be directed to the corresponding authors.

## Ethics statement

The animal study was approved by Landesdirektion Leipzig, Germany, TVV39/14. The study was conducted in accordance with the local legislation and institutional requirements.

## Author contributions

JW: Conceptualization, Formal analysis, Investigation, Methodology, Visualization, Writing – review & editing. SD: Formal analysis, Visualization, Writing – original draft. CG: Formal analysis, Investigation, Writing – review & editing. MH: Formal analysis, Investigation, Writing – review & editing. YP: Formal analysis, Investigation, Writing – review & editing. KK: Investigation, Writing – review & editing, Formal analysis. NK: Resources, Writing – review & editing, Investigation. MB: Resources, Writing – review & editing. JS: Formal analysis, Funding acquisition, Writing – review & editing, Investigation. JH: Conceptualization, Formal analysis, Funding acquisition, Project administration, Supervision, Visualization, Writing – review & editing.

## References

[1] Blüher M. Obesity: global epidemiology and pathogenesis. Nat Rev Endocrinol. (2019) 15:288–98. doi: 10.1038/s41574-019-0176-8 30814686

[2] Shoelson SE Herrero L Naaz A. Obesity, inflammation, and insulin resistance. Gastroenterology. (2007) 132:2169–80. doi: 10.1053/j.gastro.2007.03.059 17498510

[3] Loomba R Friedman SL Shulman GI. Mechanisms and disease consequences of nonalcoholic fatty liver disease. Cells. (2021) 184:2537–64. doi: 10.1016/j.cell.2021.04.015 PMC12168897 33989548

[4] Fasshauer M Blüher M. Adipokines in health and disease. Trends Pharmacol Sci. (2015) 36:461–70. doi: 10.1016/j.tips.2015.04.014 26022934

[5] Blüher M. Adipose tissue dysfunction in obesity. Exp Clin Endocrinol Diabetes. (2009) 117:241–50. doi: 10.1055/s-0029-1192044 19358089

[6] Schmitz J Evers N Awazawa M Nicholls HT Bronneke HS Dietrich A. Obesogenic memory can confer long-term increases in adipose tissue but not liver inflammation and insulin resistance after weight loss. Mol Metab. (2016) 5:328–39. doi: 10.1016/j.molmet.2015.12.001, PMID: 27110485 PMC4837291

[7] Kleinert M Clemmensen C Hofmann SM Moore MC Renner S Woods SC. Animal models of obesity and diabetes mellitus. Nat Rev Endocrinol. (2018) 14:140–62. doi: 10.1038/nrendo.2017.161 29348476

[8] Lozano I Van der Werf R Bietiger W Seyfritz E Peronet C Pinget M. High-fructose and high-fat diet-induced disorders in rats: impact on diabetes risk, hepatic and vascular complications. Nutr Metab. (2016) 13:15. doi: 10.1186/s12986-016-0074-1, PMID: 26918024 PMC4766713

[9] Sumiyoshi M Sakanaka M Kimura Y. Chronic intake of high-fat and high-sucrose diets differentially affects glucose intolerance in mice. J Nutr. (2006) 136:582–7. doi: 10.1093/jn/136.3.582, PMID: 16484528

[10] Yan RL Choi VWW Hartono T Tse IMY Tse MCL Zhou YP. Effect of lifelong sucrose consumption at human-relevant levels on food intake and body composition of C57bl/6n mice. Front Nutr. (2022) 9:1076073. doi: 10.3389/fnut.2022.1076073, PMID: 36590231 PMC9798237

[11] Montgomery MK Fiveash CE Braude JP Osborne B Brown SH Mitchell TW. Disparate metabolic response to fructose feeding between different mouse strains. Sci Rep. (2015) 5:18474. doi: 10.1038/srep18474, PMID: 26690387 PMC4686880

[12] Stephenson EJ Stayton AS Sethuraman A Rao PK Meyer A Gomes CK. Chronic intake of high dietary sucrose induces sexually dimorphic metabolic adaptations in mouse liver and adipose tissue. Nat Commun. (2022) 13:6062. doi: 10.1038/s41467-022-33840-6, PMID: 36229459 PMC9561177

[13] Oliveira LS Santos DA Barbosa-da-Silva S Mandarim-de-Lacerda CA Aguila MB. The inflammatory profile and liver damage of a sucrose-rich diet in mice. J Nutr Biochem. (2014) 25:193–200. doi: 10.1016/j.jnutbio.2013.10.006, PMID: 24445044

[14] Watson PM Commins SP Beiler RJ Hatcher HC Gettys TW. Differential regulation of leptin expression and function in a/J vs. C57bl/6j mice during diet-induced obesity. Am J Physiol Endocrinol Metab. (2000) 279:E356–65. doi: 10.1152/ajpendo.2000.279.2.E356, PMID: 10913036

[15] Jensen T Abdelmalek MF Sullivan S Nadeau KJ Green M Roncal C. Fructose and sugar: a major mediator of non-alcoholic fatty liver disease. J Hepatol. (2018) 68:1063–75. doi: 10.1016/j.jhep.2018.01.019, PMID: 29408694 PMC5893377

[16] Schultz A Neil D Aguila MB Mandarim-de-Lacerda CA. Hepatic adverse effects of fructose consumption independent of overweight/obesity. Int J Mol Sci. (2013) 14:21873–86. doi: 10.3390/ijms141121873, PMID: 24196354 PMC3856040

[17] Weiner J Rohde K Krause K Zieger K Klöting N Kralisch S. Brown adipose tissue (bat) specific Vaspin expression is increased after obesogenic diets and cold exposure and linked to acute changes in DNA-methylation. Mol Metab. (2017) 6:482–93. doi: 10.1016/j.molmet.2017.03.004, PMID: 28580279 PMC5444018

[18] von Essen G Lindsund E Maldonado EM Zouhar P Cannon B Nedergaard J. Highly recruited Brown adipose tissue does not in itself protect against obesity. Mol Metab. (2023) 76:101782. doi: 10.1016/j.molmet.2023.101782, PMID: 37499977 PMC10432997

[19] Galarraga M Campion J Munoz-Barrutia A Boque N Moreno H Martinez JA. Adiposoft: automated software for the analysis of white adipose tissue cellularity in histological sections. J Lipid Res. (2012) 53:2791–6. doi: 10.1194/jlr.D023788, PMID: 22993232 PMC3494244

[20] Matyash V Liebisch G Kurzchalia TV Shevchenko A Schwudke D. Lipid extraction by methyl-Tert-butyl ether for high-throughput Lipidomics. J Lipid Res. (2008) 49:1137–46. doi: 10.1194/jlr.D700041-JLR200, PMID: 18281723 PMC2311442

[21] Leopold J Engel KM Prabutzki P Schiller J. Combined use of MALDI-TOF mass spectrometry and (31)P NMR spectroscopy for the analysis of (Phospho) lipids. Methods Mol Biol. (2023) 2625:183–200. doi: 10.1007/978-1-0716-2966-6_17, PMID: 36653644

[22] Popkova Y Meusel A Breitfeld J Schleinitz D Hirrlinger J Dannenberger D. Nutrition-dependent changes of mouse adipose tissue compositions monitored by NMR, MS, and chromatographic methods. Anal Bioanal Chem. (2015) 407:5113–23. doi: 10.1007/s00216-015-8551-3, PMID: 25724368

[23] Schröter J Fülöp A Hopf C Schiller J. The combination of 2,5-Dihydroxybenzoic acid and 2,5-Dihydroxyacetophenone matrices for unequivocal assignment of phosphatidylethanolamine species in complex mixtures. Anal Bioanal Chem. (2018) 410:2437–47. doi: 10.1007/s00216-018-0926-9, PMID: 29445834

[24] Popkova Y Dannenberger D Schiller J Engel KM. Differences in the lipid patterns during maturation of 3t3-L1 adipocytes investigated by thin-layer chromatography, gas chromatography, and mass spectrometric approaches. Anal Bioanal Chem. (2020) 412:2237–49. doi: 10.1007/s00216-019-02243-w, PMID: 31797017

[25] Zieger K Weiner J Krause K Schwarz M Kohn M Stumvoll M. Vaspin suppresses cytokine-induced inflammation in 3t3-L1 adipocytes via inhibition of Nfkappab pathway. Mol Cell Endocrinol. (2018) 460:181–8. doi: 10.1016/j.mce.2017.07.022, PMID: 28756250

[26] Zhang WX Fan J Ma J Rao YS Zhang L Yan YE. Selection of suitable reference genes for quantitative real-time Pcr normalization in three types of rat adipose tissue. Int J Mol Sci. (2016) 17:968. doi: 10.3390/ijms17060968, PMID: 27338366 PMC4926500

[27] Fan X Yao H Liu X Shi Q Lv L Li P. High-fat diet alters the expression of reference genes in male mice. Front Nutr. (2020) 7:589771. doi: 10.3389/fnut.2020.589771, PMID: 33330591 PMC7732482

[28] Weisberg SP McCann D Desai M Rosenbaum M Leibel RL Ferrante AW. Obesity is associated with macrophage accumulation in adipose tissue. J Clin Invest. (2003) 112:1796–808. doi: 10.1172/Jci200319246 14679176 PMC296995

[29] Xu HY Barnes GT Yang Q Tan Q Yang DS Chou CJ. Chronic inflammation in fat plays a crucial role in the development of obesity-related insulin resistance. J Clin Invest. (2003) 112:1821–30. doi: 10.1172/Jci200319451, PMID: 14679177 PMC296998

[30] Kern PA Ranganathan S Li C Wood L Ranganathan G. Adipose tissue tumor necrosis factor and Interleukin-6 expression in human obesity and insulin resistance. Am J Physiol Endocrinol Metab. (2001) 280:E745–51. doi: 10.1152/ajpendo.2001.280.5.E745 11287357

[31] Hotamisligil GS Shargill NS Spiegelman BM. Adipose expression of tumor-necrosis-factor-alpha - direct role in obesity-linked insulin resistance. Science. (1993) 259:87–91. doi: 10.1126/science.7678183, PMID: 7678183

[32] Sartipy P Loskutoff DJ. Monocyte chemoattractant protein 1 in obesity and insulin resistance. Proc Natl Acad Sci U S A. (2003) 100:7265–70. doi: 10.1073/pnas.1133870100, PMID: 12756299 PMC165864

[33] Hotamisligil GS. Inflammation and metabolic disorders. Nature. (2006) 444:860–7. doi: 10.1038/nature05485 17167474

[34] Kahn BB Flier JS. Obesity and insulin resistance. J Clin Invest. (2000) 106:473–81. doi: 10.1172/JCI10842, PMID: 10953022 PMC380258

[35] Zeng H Qin H Liao M Zheng EZ Luo XQ Xiao AH. Cd36 promotes De novo lipogenesis in hepatocytes through Insig2-dependent Srebp1 processing. Mol Metab. (2022) 57:101428. doi: 10.1016/j.molmet.2021.101428, PMID: 34974159 PMC8810570

[36] Chen YL Zhang J Cui WG Silverstein RL. Cd36, a signaling receptor and fatty acid transporter that regulates immune cell metabolism and fate. J Exp Med. (2022) 219:e20211314. doi: 10.1084/jem.20211314, PMID: 35438721 PMC9022290

[37] Wilson CG Tran JL Erion DM Vera NB Febbraio M Weiss EJ. Hepatocyte-specific disruption of Cd36 attenuates fatty liver and improves insulin sensitivity in Hfd-fed mice. Endocrinology. (2016) 157:570–85. doi: 10.1210/en.2015-1866, PMID: 26650570 PMC4733118

[38] Rumessen JJ Gudmand-Hoyer E. Absorption capacity of fructose in healthy adults. Comparison with sucrose and its constituent monosaccharides. Gut. (1986) 27:1161–8. doi: 10.1136/gut.27.10.1161, PMID: 3781328 PMC1433856

[39] Hirahatake KM Meissen JK Fiehn O Adams SH. Comparative effects of fructose and glucose on lipogenic gene expression and intermediary metabolism in Hepg2 liver cells. PLoS One. (2011) 6:e26583. doi: 10.1371/journal.pone.0026583, PMID: 22096489 PMC3214012

[40] Mayes PA. Intermediary metabolism of fructose. Am J Clin Nutr. (1993) 58:754S–65S. doi: 10.1093/ajcn/58.5.754S 8213607

[41] Lanaspa MA Sanchez-Lozada LG Choi YJ Cicerchi C Kanbay M Roncal-Jimenez CA. Uric acid induces hepatic steatosis by generation of mitochondrial oxidative stress: potential role in fructose-dependent and -independent fatty liver. J Biol Chem. (2012) 287:40732–44. doi: 10.1074/jbc.M112.399899, PMID: 23035112 PMC3504786

[42] Jin R Willment A Patel SS Sun X Song M Mannery YO. Fructose induced Endotoxemia in pediatric nonalcoholic fatty liver disease. Int J Hepatol. (2014) 2014:560620:1–8. doi: 10.1155/2014/560620, PMID: 25328713 PMC4195259

[43] Jegatheesan P Beutheu S Ventura G Sarfati G Nubret E Kapel N. Effect of specific amino acids on hepatic lipid metabolism in fructose-induced non-alcoholic fatty liver disease. Clin Nutr. (2016) 35:175–82. doi: 10.1016/j.clnu.2015.01.021, PMID: 25736031

[44] Kihara A. Very long-chain fatty acids: elongation, physiology and related disorders. J Biochem. (2012) 152:387–95. doi: 10.1093/jb/mvs105 22984005

[45] Naganuma T Kihara A. Two modes of regulation of the fatty acid elongase ELOVl6 by the 3-ketoacyl-CoA reductase KAR in the fatty acid elongation cycle. PLoS One. (2014) 9:e101823. doi: 10.1371/journal.pone.0101823, PMID: 25003994 PMC4086937

[46] Jiang L Wang Q Yu Y Zhao F Huang P Zeng R. Leptin contributes to the adaptive responses of mice to high-fat diet intake through suppressing the Lipogenic pathway. PLoS One. (2009) 4:e6884. doi: 10.1371/journal.pone.0006884, PMID: 19727392 PMC2731220

[47] Guiu-Jurado E Auguet T Berlanga A Aragones G Aguilar C Sabench F. Downregulation of De novo fatty acid synthesis in subcutaneous adipose tissue of moderately obese women. Int J Mol Sci. (2015) 16:29911–22. doi: 10.3390/ijms161226206, PMID: 26694359 PMC4691149

[48] Garrido D Rubin T Poidevin M Maroni B Le Rouzic A Parvy JP. Fatty acid synthase cooperates with glyoxalase 1 to protect against sugar toxicity. PLoS Genet. (2015) 11:e1004995. doi: 10.1371/journal.pgen.1004995, PMID: 25692475 PMC4334898

[49] Schwarz JM Noworolski SM Wen MJ Dyachenko A Prior JL Weinberg ME. Effect of a high-fructose weight-maintaining diet on lipogenesis and liver fat. J Clin Endocr Metab. (2015) 100:2434–42. doi: 10.1210/jc.2014-3678, PMID: 25825943 PMC4454806

[50] Cohen CC Li KW Alazraki AL Beysen C Carrier CA Cleeton RL. Dietary sugar restriction reduces hepatic De novo lipogenesis in adolescent boys with fatty liver disease. J Clin Invest. (2021) 131:e150996. doi: 10.1172/JCI150996, PMID: 34907907 PMC8670836

[51] Schwarz JM Noworolski SM Erkin-Cakmak A Korn NJ Wen MJ Tai VW. Effects of dietary fructose restriction on liver fat, De novo lipogenesis, and insulin kinetics in children with obesity. Gastroenterology. (2017) 153:743–52. doi: 10.1053/j.gastro.2017.05.043, PMID: 28579536 PMC5813289

