# Supplementary material for: Differential expression of immunoregulatory cytokines in adipose tissue and liver in response to high fat and high sugar diets in female mice
Source: Front Nutr. 2023 Nov 3;10:1275160. doi: 10.3389/fnut.2023.1275160 (PMC10655005; doi:10.3389/fnut.2023.1275160)
Supplement: Supplementary file 1 [file Data_Sheet_1.pdf]

Supplementary material

Differential expression of immuno-regulatory cytokines in adipose tissue and liver in response to high fat and high sugar diets in female mice.

Juliane Weiner, Sebastian Dommel, Claudia Gebhardt, Martha Hanschkow, Yulia Popkova, Kerstin Krause, Nora Klöting, Matthias Blüher, Jürgen Schiller, John T. Heiker

Supplementary Table 1: Primers used in this study.

Gene Name	Forward Primer	Reverse Primer
Acc1	TACAGGATGGTTTGGCCTTT	CAAATTCTGCTGGAGAAGCC
Alk7	CCTTGCGGCAGGACTGAAG	GACCAAGTCGAGGGGCATTTG
Bmp8b	TCCACCAACCACGCCACTAT	CAGTAGGCACACAGCACACCT
Cd36	TGGAGCTGTTATTGGTGCAG	TGGGTTTTGCACATCAAAGA
Cox7a1	AGAAAACCGTGTGGCAGAGA	CAGCGTCATGGTCAGTCTGT
Cpt1a	TGGATGGCTATGGTCAAGGT	TCTCCCTCCTTCATCAGTGG
Fasn	CTCGCTTGTCGTCTGCCT	TTGGCCCAGAACTCCTGTAG
Glo1	CCTGATGACGGGAAAATGAAAG	GCCGTCAGGGTCTTGAATGA
Hsl	CTGCCCAGGATTGGATGGTT	CGCTGAGGCTTTGATCTTGC
Il1b	TGCCACCTTTTGACAGTGATG	AAGGTCCACGGGAAAGACAC
Il6	ACCTGGAGTACATGAAGAACA	TTGGAAATTGGGGTAGGAAG
Mcp1	GCCCCACTCACCTGCTGCTACT	CCTGCTGCTGGTGATCCTCTTGT
Pgc1a	CTTTTGTGGACGGAAGCAAT	GAGTCTTGGGAAAGGACACG
Pparg	CGTGAAGCCCATCGAGGACATC	TGGAGCAGGGGGTGAAG
Scd1	GAGGCCTGTACGGGATCATA	CAGCCGAGCCTTGTAAGTTC
Tbx1	GGCAGGCAGACGAATGTTC	TTGTCATCTACGGGCACAAAG
Tmem26	AGGGGCTTCCTTAGGGTTTTC	CCGTCTTGGATGAAGAAGCTG
Ucp1	CCGAAACTGTACAGCGGTCT	CCGAGAGAGGCAGGTGTTTC

Supplementary Figure S1

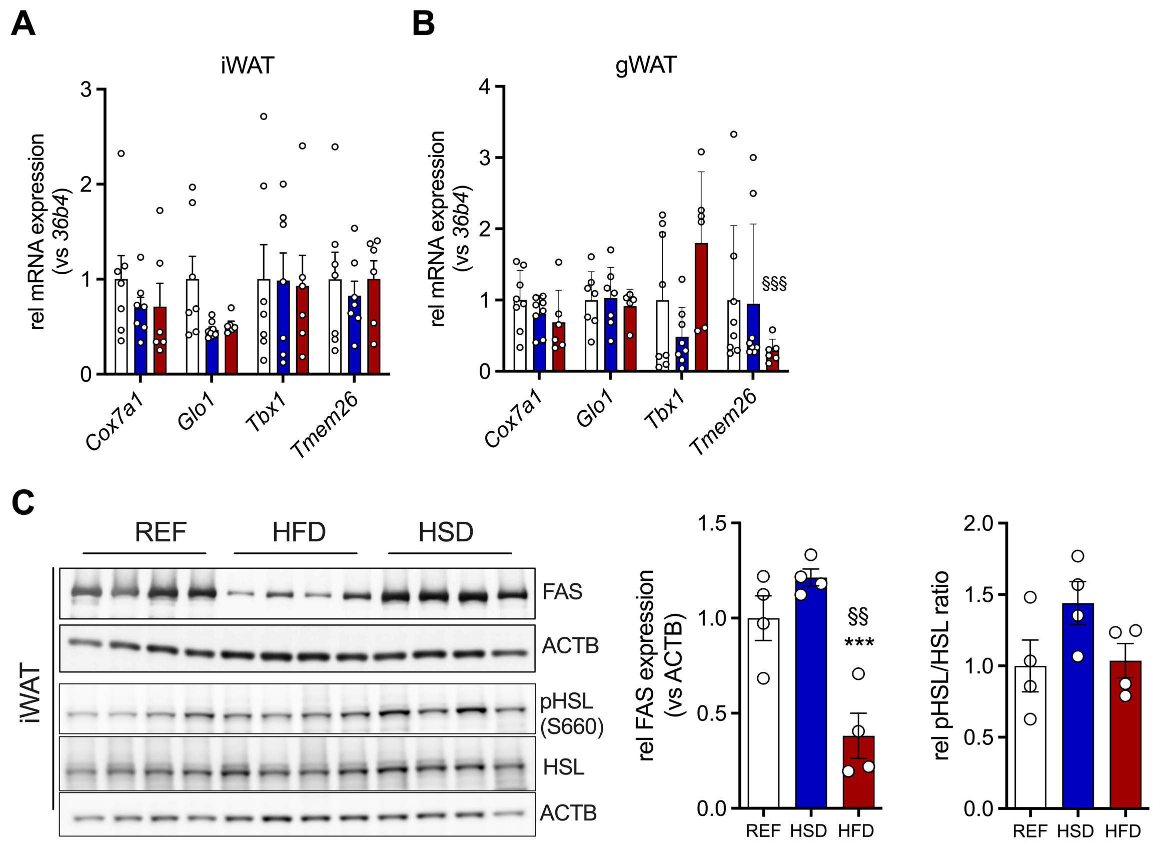

Supplementary Figure S1. Gene and protein expression in WAT depots in response to twelve weeks of HSD and HFD feeding. (A, B) The expression of thermogenic genes *Cox7a1, Glo1, Tbx1* and *Tmem26* in iWAT (A) and gWAT (B) of HFD- and HSD-fed and control mice. (C) Western blot analysis (left) of HSL phosphorylation (pHSL (Ser660) and HSL) as well as FAS expression in iWAT (n=4/group). Densitometric analyses of FAS expression normalized for bActin (middle) and HSL-phosphorylation (right). Color coding for all graphs: black - control diet; blue - HSD, red - HFD. Data are presented as mean ± SEM, with N=6-8 mice per group. Statistical significance was tested by 2-way ANOVA with Tukey's (A, B) multiple comparisons test, or by ANOVA with Tukey's (C) multiple comparisons test and is indicated as follows: *, vs. REF; § HSD vs. HFD; *, § p<0.05; **, §§ p<0.01; ***, §§§ p<0.001.
